# Anti-NOGO Antibody Neuroprotection in a Rat Model of NAION

**DOI:** 10.1167/tvst.10.14.12

**Published:** 2021-12-14

**Authors:** Mary A. Johnson, Zara Mehrabian, Yan Guo, Joy Ghosh, Mitchell G. Brigell, Steven L. Bernstein

**Affiliations:** 1 Department of Ophthalmology & Visual Science, University of Maryland School of Medicine, Baltimore, MD, USA; 2 Department of Anatomy and Pathology, University of Maryland School of Medicine, Baltimore, MD, USA; 3 Eventide Asset Management, LLC, Boston, MA, USA; 4 Aerpio Corporation, Belmont, MA, USA

**Keywords:** NOGO, rNAION, RGC, optic nerve, inflammation

## Abstract

**Purpose:**

To evaluate the efficacy and mechanisms of anti-NOGO receptor monoclonal antibody 11C7mAb in a rat model of nonarteritic anterior ischemic optic neuropathy (rNAION).

**Methods:**

The rNAION was induced in one eye of 20 Long-Evans rats, which were studied in 10 groups of two rats, each group containing a sham rat receiving intravitreal injections of vehicle and a treatment rat receiving intravitreal injections of 11C7mAb. Fellow eyes served as naïve controls. The rats were tested using flash electroretinograms (ERGs), flash visual evoked potentials (VEPs), and optical coherence tomography (OCT). Thirty days after induction, they were euthanized, and the eyes were prepared for immunohistochemistry (two groups), hematoxylin and eosin staining (two groups) or transmission electron microscopy (TEM; six groups).

**Results:**

Ninety-five percent of the VEP amplitude was preserved in eyes treated with 11C7mAb, versus 69% in the control eyes. Immunohistochemistry revealed a large reduction in microglia and extrinsic macrophages with axon sparing. In addition to axon preservation, TEM also showed reduced extracellular debris and only slight myelin damage compared with the vehicle-treated animals. There were no significant differences in retinal ganglion cell counts, nor was there a difference in optic nerve swelling as measured by OCT. ERGs were used to exclude eyes with retinal vascular occlusions, an occasional complication of the induction technique.

**Conclusions:**

The 11C7mAb preserves optic nerve integrity and reduces inflammation in rNAION.

**Translational Relevance:**

This study evaluates the efficacy of an anti-NOGO receptor antibody in a rat model of NAION, a disorder that currently has no universally-acknowledged treatment.

## Introduction

Nonarteritic anterior ischemic optic neuropathy (NAION) is the most common cause of new optic-nerve related vision loss in individuals over the age of 50,^1^ accounting for roughly 10,000 new cases per year in the United States.^2^^,^^3^ In this disease, the retinal ganglion cell (RGC) axons comprising the optic nerve (ON) develop sudden focal ischemia at the anterior border of the ON, with subsequent loss of RGC axons and post-stroke demyelination.^1^^,^^4^ After first eye involvement, there is a 25% likelihood over five years that the normal, fellow eye will be involved as well.^5^ There is no widely accepted effective therapy for NAION.

We have developed both rodent^6^ and primate^7^ models of NAION (rNAION, pNAION, respectively) that mimic human NAION in clinical appearance, electrophysiologic responses, and histologic and histochemical findings. In particular, we have found that both our models and human NAION are characterized in part by a marked inflammatory reaction.^8^ This inflammation occurs in the anterior ON near the junction of the retina and optic nerve in both the disease and the models. We previously have had success in preventing loss of visual function and retinal ganglion cells (RGC) in our rat model of NAION (rNAION), using intravitreal and systemically administered prostaglandin 15-deoxy 12,14 delta prostaglandin J2 (PGJ_2_) as well as using topical administration of trabodenoson.^9^ However, although delayed administration of these agents has successfully prevented damage in rNAION and pNAION (neuroprotection), it does not restore either optic nerve function or optic nerve integrity (neuroregeneration).^10^ We wished to evaluate a neuroregenerative approach in NAION treatment.

Development is a time of explosive neural growth in the central nervous system. At the conclusion of development, Nogo-A and other inhibitory proteins present in oligodendricytes and myelin are activated, preventing continued expansion of neurons.^11^ A major recognized barrier to regeneration in the central nervous system is the presence of degenerate myelin products including the 66aa fragment of NOGO-A protein, which inhibits the growth of axons after development.^12^^–^^14^ Blocking NOGO-A has been shown to markedly increase neurite sprouting and axon regrowth in spinal cord trauma^15^^,^^16^ and in stroke,^17^^–^^19^ improving function in both. The vast majority of RGCs express NOGO receptors,^20^ and intravitreal injection of NOGO-blocking compounds have been demonstrated to reduce RGC loss and to act neuroregeneratively to increase axonal sprouting in mouse glaucoma models.^21^ This may be at least partly due to the loss of blood-optic nerve barrier integrity, suggesting that local administration of a high concentration of a therapeutic in the region of the ischemic lesion may be effective. In retinal excitotoxic cell death mediated by N-methyl-D-aspartate (NMDA), NOGO-A blockade increases visual function as measured by optokinetic nystagmus.^22^ In the current study, we test the neuroprotective/regenerative effect of a monoclonal antibody to NOGO-A, 11C7mAb, in a rat model of NAION.

## Methods

### Animals

Male Long-Evans rats (225–300 g), obtained from Harlan Labs (Indianapolis, IN, USA) were kept in the University of Maryland, Baltimore, animal facility and were given food and water as desired. Animals were anesthetized for all procedures with an intraperitoneal injection of a mixture of ketamine/xylazine (80 mg/kg; 4 mg/kg). All animal protocols were approved by the University of Maryland, Baltimore, Institutional Animal Care and Use Committee and were handled in accordance with the ARVO Statement for the Use of Animals in Ophthalmic and Vision Research.

### Induction of rNAION

The rNAION was generated as described previously.^6^ Briefly, a specially designed contact lens was placed on the cornea of an anesthetized rat to visualize the retina and ON. The rNAION then was induced using an intravenous injection of rose bengal (2.5 mM), followed by laser illumination of the optic disc (500 µm spot size, 50 mW power, 9–11 seconds duration) via a frequency-doubled YAG laser (535 nm; Iridex Corporation, Mountain View, CA), coupled to a slit-lamp biomicroscope (Haag-Streit 900; Haag-Streit, Mason, OH, USA). Animal retinas were examined at two days after lesion induction (i.e., stroke) to evaluate the degree of rNAION. The extent of intraocular optic nerve edema was also determined using spectral domain-optical coherence tomography (SD-OCT) (see paragraph below). Animals exhibiting significant retinal ischemia (as compared with isolated optic nerve head edema) at this time were excluded from the study.

### Study Design

Twenty male Long-Evans rats (225–300 g) were tested in 10 groups of two rats. Each group was comprised of one control animal, and one rat receiving the experimental anti-NOGO 11C7mAb antibody (supplied by Novartis, Basel, Switzerland). This was done to minimize variability between the control and experimental groups, as there is significant variability in the model and treating an animal from each group in the same session minimizes sources of variability that are difficult to control. We induced rNAION in one eye of each rat, with the fellow eye serving as a naïve control. The induced eyes received either an intravitreal injection of 2 µL of control antibody (mAb to wheat germ agglutinin), or the 11C7mAb antibody, immediately after rNAION induction and one week later. Endotoxin associated with production of the 11C7mAb antibody was removed using additional rounds of affinity purification.

### Electrophysiology

Electrophysiology was performed and analyzed by an individual blinded to the drug assignment. Electroretinography (ERG) and flash visual evoked potentials (VEPs) were performed at baseline and one month after stroke in eight of the 10 groups of animals, per agreement with Novartis, who funded the study. ERGs were performed to exclude animals that developed postinduction retinal ischemia, an occasional side effect of rNAION induction. Baseline VEPs were recorded to insure that both eyes were similar prior to the start of any experimental procedures. Initially, the majority of eyes tested showed ERG b-wave reductions of more than 30%, consistent with occlusion of vessels in the inner retina, so the laser fluence was reduced. This resulted in none of the animals having significant losses in b-wave amplitudes, but it also resulted in induction of milder strokes. All of the animals reported here received this lower level of induction; thus, the results presented here likely represent a ceiling effect.

Animals were dark-adapted for a minimum of 12 hours before electrophysiologic procedures, and each animal re-adapted for five to 10 minutes between repetitions of the VEP to produce a stable level of dark adaptation. After anesthesia, flash VEPs were recorded first, using a 627-nm stimulus with a half band-pass of 10 nm and a radiance of −2.52 log W/sr m^2^ (or J/sr m^2^), and the minimal number of flashes necessary (typically 40–60) to produce a repeatable response; this was done to minimize light adaptation. VEPs were repeated a number of times in each eye, alternating eyes, until waveforms were repeatable. As the assigned treatment was not revealed to the equipment operator, the variation in the exact number of flashes was unlikely to contribute to bias. VEPs were recorded using gold disc skin electrodes and EC2 electrode paste (Grass Instruments, Warwick, RI). The active electrode was placed in the occupital prominence, the indifferent in the frontal (Fz) position, and the ground on the tail. VEP amplitudes were measured between N50 and the highest positive peak, whose latency changed depending on the exact level of light adaptation. For this reason, latency was not used as a parameter in this experiment. At one-month post-stroke, the ratio of the amplitude in the affected eye to the amplitude in the normal fellow eye was analyzed. In this way, each animal served as its own internal control. Following VEP measurements, animals were dark-adapted for at least 10 minutes prior to ERG recording. All rat manipulation was performed under dim red light that we determined to have minimal if any effect on the ERGs. Full-field ERGs were recorded binocularly using the standard ISCEV protocol.^23^ Microfiber active electrodes were placed on the corneas and were referenced to a skin electrode placed at the frontal scalp, an anterior location on top of the head. ERG b-wave amplitudes were measured from the trough of the a-wave to the peak of the b-wave, as by convention.

### Optical Coherence Tomography 

Optical coherence tomography (OCT) using the Heidelberg Spectralis spectral-domain OCT was performed on each eye of anesthetized animals at baseline and at two days after rNAION induction. We used a custom-made plano-convex fundus contact lens with a thin front surface (Micro-R, now commercially available through Nissel & Cantor, London, UK) to obtain retinal cross-sectional images, as well as the typical en face view. Optic nerve swelling near the optic disc was assessed as previously described.^24^ Briefly, we measured the shortest distance from the inner nuclear layer on the nasal side of the disc to the inner nuclear layer on the temporal side of the disc, using Heidelberg proprietary software (see Fig. 3). The person measuring disc diameter was blind to the treatment given the animal.

### Postmortem Analyses

Animals were euthanized at 30 days post rNAION, a time at which the vast majority (>95%) of rNAION-induced RGC loss has occurred.^25^ Deeply anesthetized animals were perfused with 4% paraformaldehyde-phosphate-buffered saline solution. After this, eyes with attached optic nerves were enucleated, punctured at the cornea and post-fixed in 4% paraformaldehyde-phosphate-buffered saline solution for 24 hours. Hematoxylin and eosin cross-section using paraffin-embedded portions was performed on a limited number of animals to evaluate overall appearance. Immunohistochemistry was performed using frozen sections embedded in optimal cutting temperature compound to evaluate the presence or absence of inflammation, viable axons and myelination. Transmission electron microscopy (TEM) was performed on two groups of animals to further document axon integrity. Optic nerve tissues used for TEM studies were fixed further in a mixture of buffered paraformaldehyde-glutaraldehyde.

Whole retinas were reacted with goat-antihuman Brn3a antibody and visualized using Cy_3_-labeled donkey antigoat secondary antibody. Quantification (using stereology) of RGC loss was performed in all animals by counting Brn3a-immunostained RGC nuclei in flat mounted retinas, using the stereoinvestigator program (MBF Bioscience; Williston, VT), which drove a motorized stage on an Nikon E800 fluorescent microscope.^10^ RGC density and cell density loss is not equally distributed across the retina; thus, estimation of RGC loss was determined by stereology, which is a systematic and statistically unbiased method that does not depend on tissue size, shape, or distribution.^26^ It is based on the principle that any RGC has an equal probability of being sampled independent of its location, size, and shape. The following measurements were used in our counts, which gives us about 87000-90000 RGC cells per naïve retina of Long-Evans rats: Counting frame width × height: 60.0 × 60.0 µm; sampling grid (X, Y): 400.0 × 400.0 µm; sampling grid area (XY): 160000 µm^2^; estimated population using user defined section thickness: 87822; total markers counted: 1976. After outlining the boundaries of the flat mounted retina at low magnification (×4) on the computer graphics display, the software placed within each subfield boundary a set of optical counting frames in a random fashion. Brn3a-immunostained RGCs were counted in optical dissectors 20 µm in depth. All analyses were performed with a Nikon Plan Apo × 60 oil objective. Cells showing an accumulation of Brn3a in the nuclei were counted with at least 300 sampling sites in each retina to ensure robustness of the data. Cell counts were performed by an individual blinded to the drug assignment.

**Table.** Antibodies

Antibody	Stains	Source	RRID	Dilution
SMI-312	Intact Axons	Abcam (mouse)	AB_448151	1:5000
IBA1	All Inflammatory Cells	Wako (rabbit)	AB_839504	1:500
Brn3a	RGC Nuclei	Santa Cruz (goat)	AB_2167511	1:500
CD68 (ED1)	Macrophages	Abcam (mouse)	AB_307338	1:500

### Analysis of Anterior Optic Nerve Inflammation

The first 1 mm of the anterior ON, including the intrascleral portion of the ONH was isolated from each animal 30 days postinduction, with the remainder of the globes saved for RGC stereology, and postfixed in 2% PFA-PBS. Serial 10-µm cross-sections were taken from both nerves and reacted with the appropriate antibodies (see Table).

### Statistics

The Wilcoxon Mann-Whitney rank sum test (unpaired data) was used to compare groups because the D'Agostino and Pearson omnibus normality test was indeterminate for the vehicle group, and the data in the 11C7mAb group were nonnormally distributed. Parametric tests require that the data be normally distributed or the tests are invalid.

## Results

Individual results from animals in groups 9 and 10, as well as results from the entire cohort, are presented below.

### VEP

**Figure 1.**
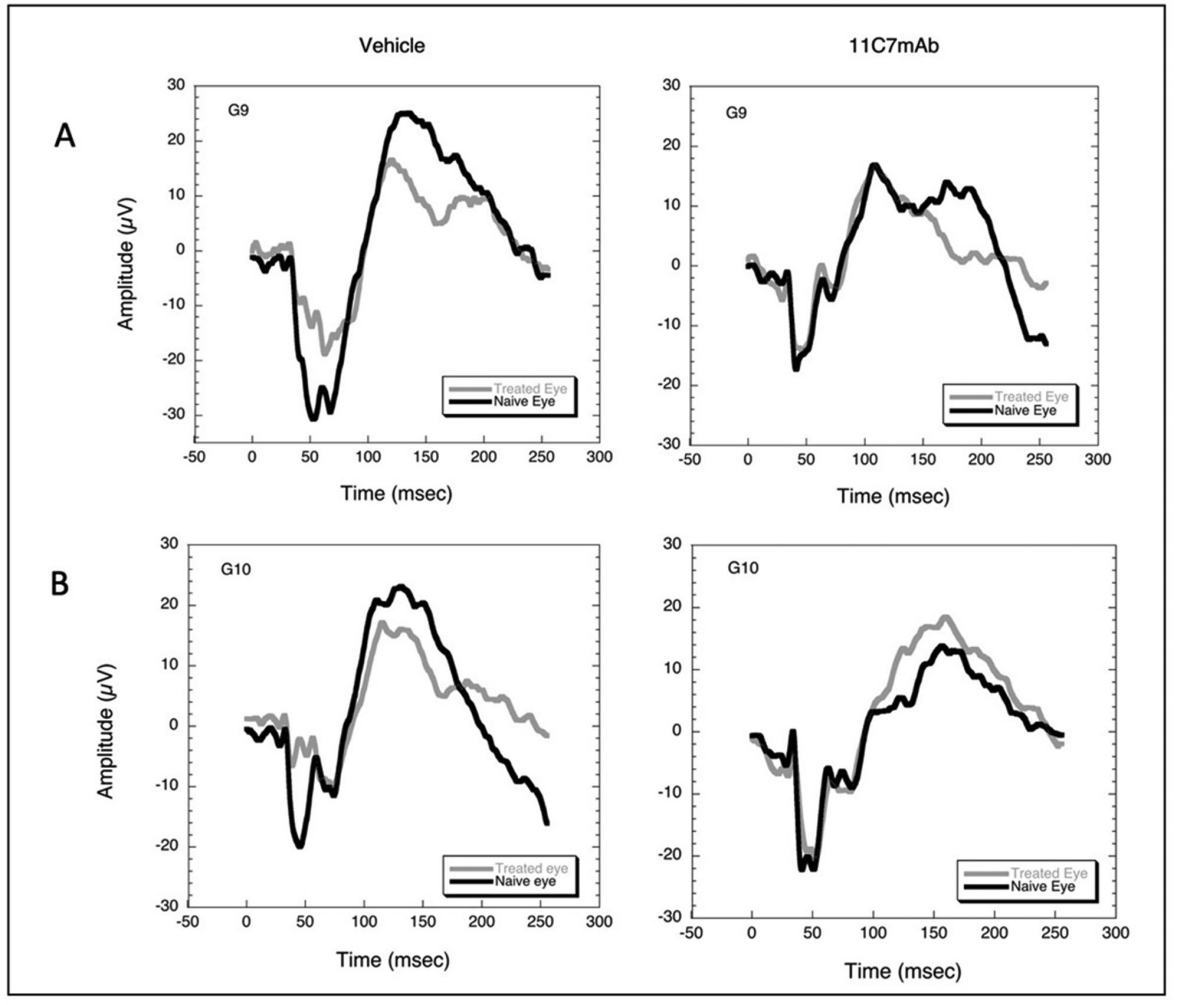
VEPs recorded 30 days after induction from four rats in groups 9 (A) and 10 (B) illustrate VEP preservation in animals that received anti-NOGO compared to animals receiving vehicle. In all panels, the *gray lines* represent the VEP recorded from the treatment eye, and the *black line* is the VEP recorded from the naïve, fellow eye.

**Figure 2.**
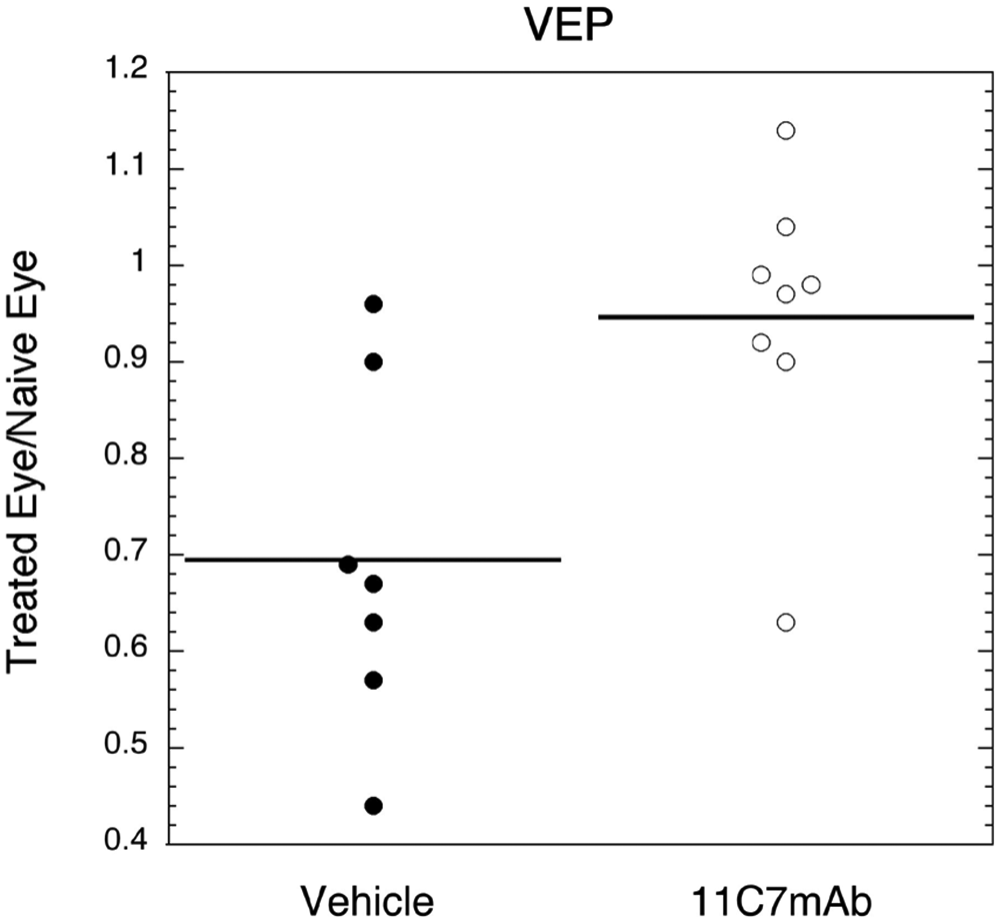
VEPs recorded at 30 days after rAION for eight groups of animals are consistent with axon preservation in the treatment group. The percent of amplitude remaining was calculated as the ratio of VEP amplitudes of the treatment eye to the naïve eye. Sixty-nine percent of the VEP was preserved (median 63%), on average, for the animals treated with vehicle, whereas 95% of the VEP was preserved in animals treated with anti-NOGO (median 97%, *p* = 0.017).

Ninety-five percent of the VEP was preserved in animals treated with 11C7mAb (see Figs. 1 and 2).

Visual function as measured by the ratio of VEP amplitudes in the treatment eye to the fellow eye at 30 days after stroke was significantly better in the eye receiving anti-NOGO than in the vehicle eye (*p* = 0.017; Fig. 2).

### OCT

**Figure 3.**
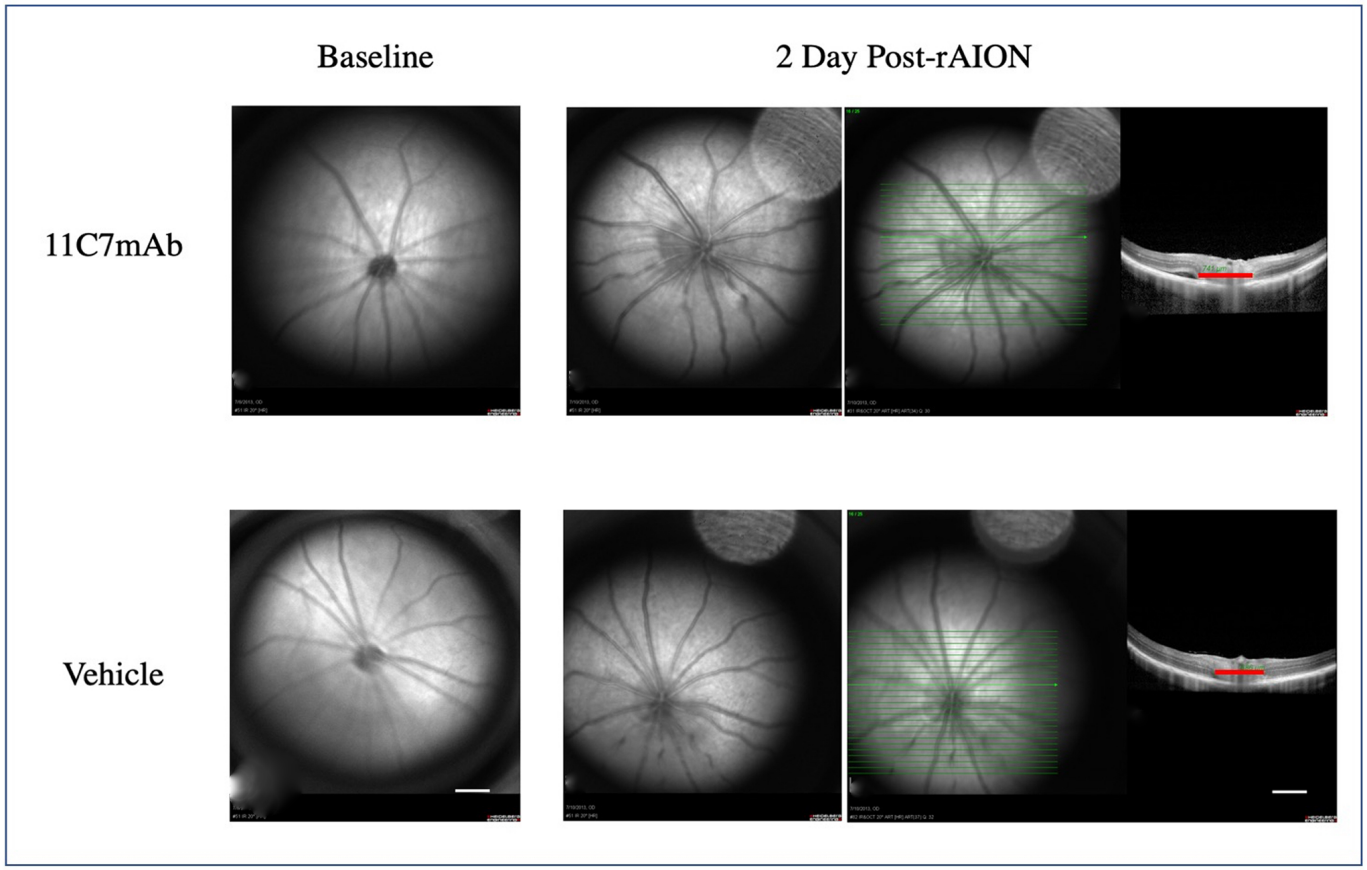
The 11C7mAb did not significantly affect ON swelling (compared with vehicle, *p* = 0.30). The *red lines* in the rightmost figures are measurements of ON disc diameter, measured as the shortest distance from the inner nuclear layer (INL) on the nasal side of the disc to the INL on the temporal side of the disc.

**Figure 4.**
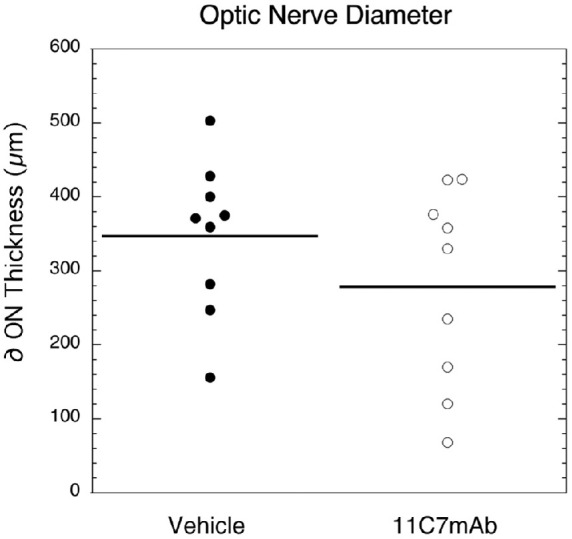
The mean change in OCT-measured expansion of the optic disc diameter from baseline to two days after induction of NAION. Eyes injected with vehicle had a mean ON diameter ± 1 SEM of 347 ± 34.5 µm). Optic discs from eyes injected with 11C7 mAb (mean diameter = 278 ± 44.7 µm) were not statistically thinner than eyes injected with vehicle (*p* = 0.30).

In contrast to what we previously have reported for PGJ_2_^10^ ON swelling did not decrease with 11C7mAb (vs. vehicle, *p* = 0.30; Figs. 3 and 4). ON swelling was measured as the shortest distance from the inner nuclear layer (INL) on the nasal side of the disc to the INL on the temporal side of the disc (see red lines in Fig. 3), using Heidelberg proprietary software.

### RGC Counts

**Figure 5.**
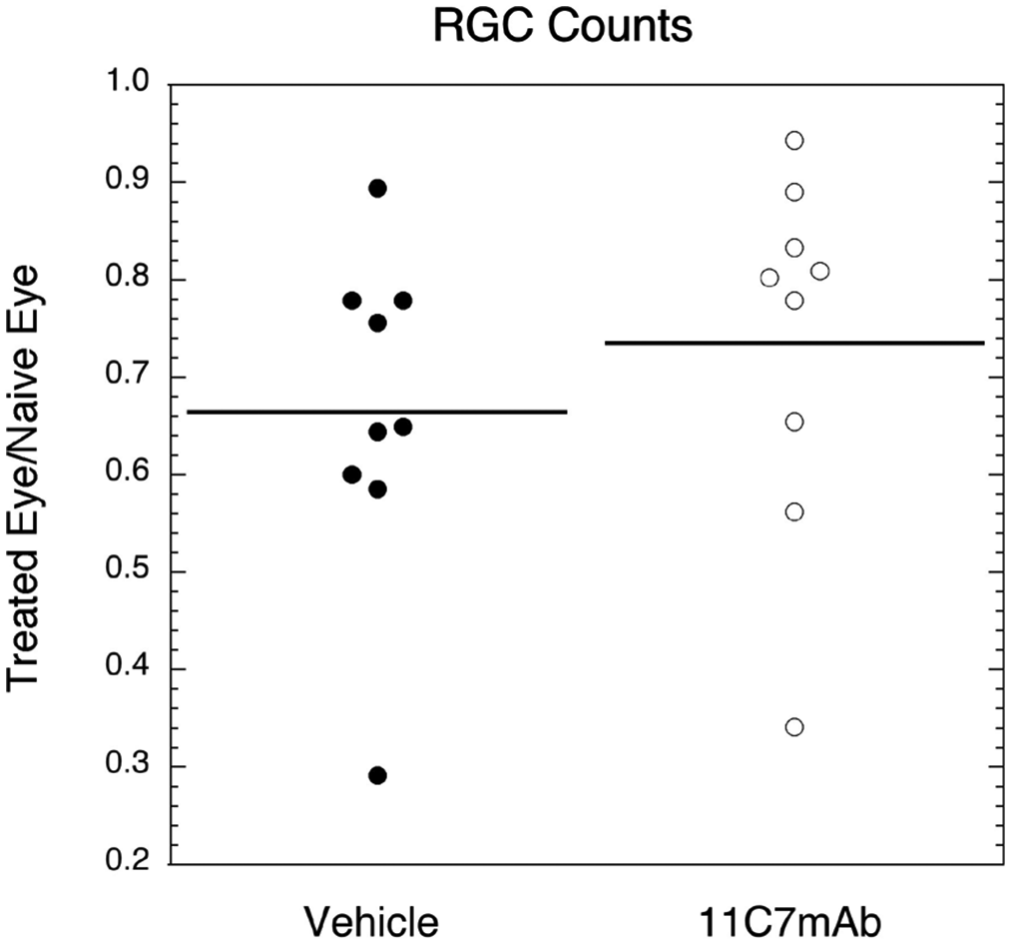
Percent of remaining RGCs of the treatment eyes compared with counts from the fellow, naïve eyes. There was no statistical difference between groups. On average, 65.0% of the ganglion cells survived in the vehicle eye (median 64.7%) and 75.0% in the eye injected with 11C7mAb (median 80.9%).

RGC numbers, measured 30 days after rNAION induction, were not statistically different between the vehicle and treatment groups. A mean of 65.0% of the ganglion cells survived in the vehicle eye (median 64.7%), and 75.0% in the eye injected with 11C7mAb (median 80.9%). The 11C7mAb did not perform statistically better than vehicle (*p* = 0.132; Fig. 5).

### Immunohistochemistry

**Figure 6.**
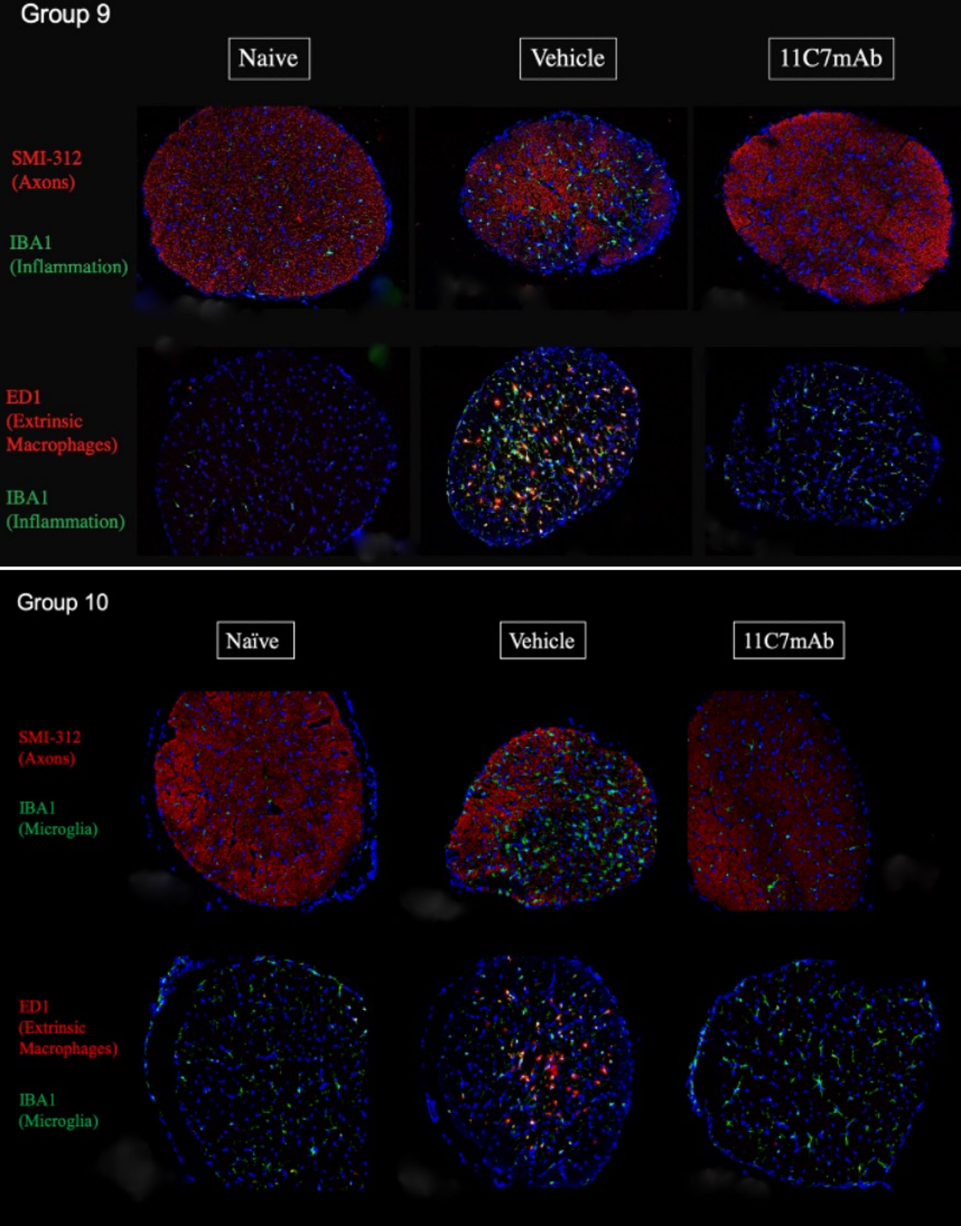
Immunohistochemistry of SMI-312(+) axon bundles, IBA1(+) microglia, and ED1(+) extrinsic macrophages in naïve (*left*), vehicle-treated (*middle*), and 11C7mAb-treated (*right*) optic nerve cross-sections. Results from groups 9 (top) and 10 (bottom) show a large inflammatory response and dropout of axons 30 days after induction in the vehicle-treated eyes, but with what appears to be much less inflammation and more axon preservation in eyes treated with 11C7mAb. Note that many of the microglia in the group 10, 11C7mAb eye appear to be ramified (resting).

Confocal microscopy of ON cross-sections obtained immediately posterior to the lamina cribrosa was performed on the rats in groups 9 and 10 (Fig. 6). Naïve eyes revealed an even distribution of axon bundles and microglia, and few extrinsic macrophages. Thirty days post rNAION induction, vehicle-treated rNAION-induced ONs revealed a regional loss of SMI-312(+) (intact) axons, with some areas appearing to show complete axonal loss. Vehicle-treated eyes showed large influxes of ED1(+) and IBA1(+) cells, suggesting macrophage invasion and microglia activation, respectively, particularly in areas of axon loss. In contrast, rNAION-induced eyes treated with 11C7mAb showed a more normal SMI312(+) staining pattern, with what appeared to be far fewer macrophages. Microglia in these ONs numbered far fewer than in the vehicle ON and were ramified (rest – not activated). Immunohistochemical results were consistent among groups. Hematoxylin and Eosin staining of ON cross-sections (not shown) on selected animals was in agreement with SMI-312(+) staining, showing less disruptive inflammatory cell infiltration in 11C7mAb-treated animals than in vehicle-treated animals.

### TEM-Ultrastructural Analysis

**Figure 7.**
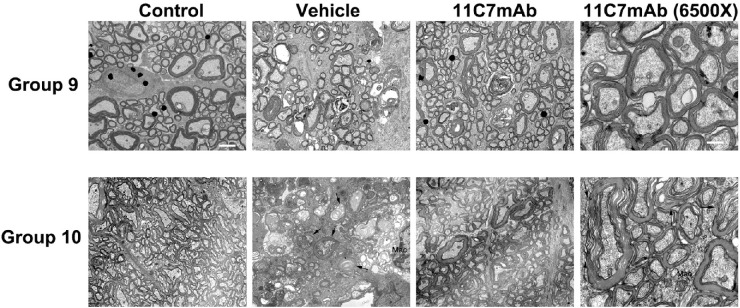
TEM comparison of naïve and treated ONs. All photomicrographs were taken at 1650× magnification except for the last column, which were taken at 6500×. The top micrographs show the results from one group of rats (Group 9), whereas the bottom micrographs show data from a different set of animals (Group 10). The naïve animal nerve is comprised of small-, medium-, and occasionally large-diameter, myelinated axons. There is no unwinding, suggesting that the myelin changes seen in the infarct/treated nerves are not due to post-perfusion artifact. There is no myelin damage and no unpacking (decreased density). The *black dots* are uranyl acetate artifacts. The vehicle-treated, induced eyes show many degenerating axons, with interstitial debris and loss of many of the large-diameter axons. Axoplasmic damage was apparent, especially in the larger axons. Another prominent feature was the engulfment of the debris in the axon fields, which was nearly absent in the 11C7mAb groups. *Arrows* point to degenerating, dead, and dying axons, and *arrowheads* show regional areas of axonal loss. The presence of a macrophage (*Mac*) is indicated. In contrast, the 11C7mAb-treated eye reveals sparing of many large-caliber axons, with reduced extracellular debris, compared with the vehicle-treated ON. The myelin is slightly damaged compared to the severe myelin damage seen in vehicle treatment, with focal unwinding (*arrows*). *Scale bar* (magnification × 1650): 2 µm. *Scale bar* (magnification × 6500): 500 nm.

Electron microscopy revealed relative axon sparing in the eyes treated with 11C7mAb compared with vehicle (Fig. 7).

## Discussion

The optic nerves with rNAION show greater preservation of structure and function in animals treated with 11C7mAb than in animals receiving vehicle. VEP amplitude was largely preserved in these subjects, by 95% versus 65% in vehicle-treated animals. Ultrastructural analyses were consistent with the VEP results, showing well-preserved medium and small axons and only mild disruption of myelin in some of the large axons, with very small amounts of axon loss. Immunohistochemistry and TEM confirmed minor loss of axons in the nerves from these eyes. There were few activated microglia. Some of the microglia appeared to be in a phase halfway between nonactivated (ramified) and activated (globoid). There were few extrinsic ED1(+) macrophages in these nerves. Surprisingly, RGC numbers in 11C7mAb-treated eyes were not significantly greater than vehicle-treated eyes, even though much of the VEP amplitude was preserved by the 11C7mAb treatment. Our results are consistent with the improvement of function without neurogenesis, as reported by other investigators using NOGO blocking antibodies.^27^

In a mouse model of NMDA-induced excitotoxicity, Mdzomba and colleagues^28^ found that a single injection of 11C7mAb, administered two days after NMDA injection, resulted in preservation of visual function determined by optokinetic testing in mice even though there were no differences in RGC numbers between experimental and sham eyes. Visual responses in both groups were equivalently depressed after NMDA injection and then continuously recovered for about the first 10 to 15 days after 11C7mAb or sham injection. However, the 11C7mAb animals recovered function to a greater extent and maintained that recovery until 40 days after 11C7mAb injection, at which point the animals were euthanized.

In spinal cord injuries, functional preservation by anti-NOGO antibodies is attributed to regrowth of optic nerve neurons and myelin regeneration by newly formed oligodendrocytes.^27^ In cortical stroke, anti-NOGO treatment and recovery of function also has been associated with revascularization in the ischemic zone penumbra surrounding the area of infarct.^29^ An additional recently recognized effect of 11C7mAb is its role in reducing neuroinflammation. In a mouse model of excitotoxicity, TNF-α expression in RGCs and Muller cell end feet was reduced by 66% in animals injected with 11C7mAb, although there were no differences in the number of Iba-positive cells.^28^ In our rat model of NAION, we found an apparent large reduction in the number of macrophages and microglia in the optic nerves of 11C7mAb-treated animals. NOGO-A has been shown to have a major role in recruiting macrophages.^30^ Nevertheless, strong reductions in neuroinflammation were apparent in both studies.

There is large variability in the amount of damage induced in rNAION model, and the causes of this are the subject of a recent report.^31^ This variability likely has many sources, including the fast- and short-lived peak dye concentration time during circulation, subtle variations in the disc morphology and angle of the laser impact of each animal treated, and individual genetic variations in outbred animals. Complicating this issue is the fact that rats and other rodents have an optic disc dominated by large arteries and veins. Thus they can develop retinal vein and artery occlusions, the probability of which increases with the strength of induction. In this study, use of ERGs excluded those animals with evidence of retinal vascular occlusion. Consequently, the study likely displays a ceiling effect that may underestimate the size of the differences among groups.

Unlike 11C7mAB, PGJ_2_ profoundly reduces optic disc edema,^32^^,^^33^ and neuroprotection works largely through this mechanism.^34^ Thus PGJ_2_ and 11C7mAB appear to protect and preserve the optic nerve in rNAION via very different mechanisms, suggesting a useful synergistic approach using two compounds with markedly different mechanisms. These findings may be important for an eventual clinical treatment to prevent or limit optic nerve damage and consequent visual loss in human NAION.
